# Effects of combinations of curcumin, linalool, rutin, safranal, and thymoquinone on glucose/serum deprivation-induced cell death

**Published:** 2013

**Authors:** Bagher Alinejad, Ahmad Ghorbani, Hamid Reza Sadeghnia

**Affiliations:** 1 ***Department of Pharmacology, School of Medicine, Mashhad University of Medical Sciences, Mashhad, I. R. Iran***; 2 ***Department of Pharmacolog and Applied Medicine, Institute of Medical Plants, ACECR, Tehran, I. R. Iran***; 3 ***Pharmacological Research Center of Medicinal Plants, School of Medicine, Mashhad University of Medical Sciences, Mashhad, I. R. Iran***; 4 ***Neuroscience Research Center, School of Medicine, Mashhad University of Medical Sciences, Mashhad, I. R. Iran***

**Keywords:** Curcumin, Linalool, PC12, Rutin, Safranal, Thymoquinone

## Abstract

**Objective:** Several phytochemical agents have been known to exhibit a neuroprotective effect. Among them, curcumin, linalool, rutin, safranal, and thymoquinone were widely investigated and neuroprotective activity of each of them was shown by several studies. This work was planned to investigate whether different combinations of them could induce better neuroprotective effect against glucose/serum deprivation (GSD)-induced cytotoxicity.

**Materials and Methods:** PC12 cells were cultivated for 8 h in GSD condition in both the absence and presence of curcumin, linalool, rutin, safranal, thymoquinone, or combinations of them. At the end of the experiment, the cell viability was determined using MTT assay.

**Results: **The cells cultured in GSD condition showed a significant decrease in viability (28±1%) as compared with those cultured in standard condition (100±2%). In the presence of curcumin (10 µg/ml), linalool (16 µg/ml), rutin (200 µg/ml), safranal (50 µg/ml), and thymoquinone (1 µg/ml), the cell viability increased to 69±3.4% (p<0.001), 44±1.4% (p<0.01), 64±0.5% (p<0.001), 49±2% (p<0.001), and 70±3.2% (p<0.001), respectively. When different combinations of the agents were tested, the best cytoprotective activity was obtained from safranal + curcumin + thymoquinone (97±5%, p<0.01 *vs.* untreated cells).

**Conclusions:** The present study demonstrated that a combination of safranal + curcumin + thymoquinone can block GSD-induced cell death and has the potential to be considered for management of cerebral ischemia and neurodegenerative diseases.

## Introduction

Deprivation of cultured neurons from glucose and serum is a reliable *in vitro* model for development of new products for management of cerebral ischemia and neurodegenerative disorders (Ghorbani et al., 2011 ▶; Mousavi et al., 2010a ▶; Sadeghnia et al., 2012 ▶). Cerebral ischemia is caused by restriction of blood flow to the brain, resulting in deficient supply of oxygen, glucose, and serum, thus leading to neuronal damage (Broughton et al., 2009 ▶). Currently, limited treatments exist for the management of neuronal damage following cerebral ischemia. Therefore, the search for new therapeutics is continued. Phytochemicals have always been good candidates to find new therapeutic drugs. Several phytochemical agents have been known to protect neurons against ischemic insult. Among them, curcumin, linalool, rutin, safranal, and thymoquinone are widely investigated and neuroprotective effects of each of them were shown by several studies.

Curcumin (diferuloylmethane) is the major active constituent of *Curcuma longa*, a spice employed as a coloring and flavoring supplement in many foods. Evidence suggests that curcumin has antioxidant, anti-inflammatory, neuromodulatory, and cytoprotective effects (Mendonca et al., 2012 ▶; Park et al., 2008 ▶; Rui et al., 2008 ▶; Tamaddonfard, 2012 ▶; Tamaddonfard et al., 2012 ▶).

Linalool, a plant-derived monoterpene alcohol, is a component of a number of essential oils including lavender, coriander, and sweet basil. The beneficial effect of linalool on nervous disorders is well documented (Batista et al., 2010 ▶; Devi et al., 2007 ▶; Elisabetsky et al., 1995 ▶). Rutin, a flavonoid constituent of foods and plant-based beverages, has several pharmacological properties including cytoprotective (Janbaz et al., 2002 ▶), anticonvulsant (Nassiri-Asl et al., 2008 ▶), and strong antioxidant (La Casa et al., 2000 ▶) activities. Furthermore, neuroprotective effect of rutin has been shown against neuronal death induced by focal ischemia and by ischemia-reperfusion injury (Khan et al., 2009 ▶; Pu et al., 2007 ▶).

Safranal, a monoterpene aldehyde, is the main constituent of the essential volatile oil in *Crocus sativus* and responsible for the characteristic aroma and odor of this plant (Tarantilis et al., 1995 ▶). Recently, it has been demonstrated that safranal and *C. sativus* have neuroprotective effects *in vitro* and *in vivo* studies (Fukui et al., 2011 ▶; Hosseinzadeh et al., 2005 ▶; Hosseinzadeh and Sadeghnia, 2007 ▶; Mousavi et al., 2010b ▶; Mousavi and Bathaie, 2011 ▶).

Thymoquinone is the natural main constituent of the volatile oil in *Nigella sativa* seeds. The protective effect of *N. sativa* and thymoquinone against glucose/serum deprivation (GSD)-induced neuronal damage has also been reported by Mousavi and colleague (2010a) ▶. Based on the evidence mentioned above, we hypothesized that combinations of curcumin, linalool, rutin, safranal, and thymoquinone may have higher neuroprotective effect and can be used as therapeutic agent for cerebral ischemia and neurodegenerative diseases. Therefore, the present work was carried out to investigate whether different combinations of these phytochemicals are capable to protect neuronal cells against GSD-induced cell death.

## Materials and Methods

**Cell lines and chemicals**

PC12, a rat pheochromocytoma-derived cell line, was obtained from Pasteur Institute (Tehran, Iran). High glucose (4.5 g/L) Dulbecco’s Modified Eagles Medium (DMEM), glucose-free DMEM and fetal bovine serum were purchased from Gibco (Carlsbad, CA). Penicillin, streptomycin, and 3-(4,5-Dimethyl-2-thiazolyl)-2,5-Diphenyl-2H-tetrazolium bromide (MTT) were obtained from Sigma (USA).

**Cell culture and treatment**

PC12 cells were cultivated in a standard medium (high-glucose DMEM supplemented with 10% fetal bovine serum). The cells at sub-confluent stage were harvested using trypsin and aliquots of 100 µl of cell suspension (5×10^4^ cells/ml) seeded in 96-well plate. Twenty-four hours later, the standard medium was replaced by glucose- and serum-free DMEM containing curcumin (1-50 µg/ml), linalool (4-32 µg/ml), rutin (50-400 µg/ml), safranal (50-400 µg/ml), thymoquinone (1-50 µg/ml), or combinations of them. These concentrations were chosen according to our previous works on linalool, rutin, and safranal (unpublished studies) as well as literature reports on neuroprotective effects of curcumin and thymoquinone (Mousavi et al., 2010a ▶; Park et al., 2008 ▶). The cells were further incubated in the GSD condition for 8 h at 37 ˚C and 5% CO_2_.

**Cell viability assay**

The cell viability was determined using MTT assay as previously described (Hadjzadeh et al., 2006 ▶; Mortazavian and Ghorbani, 2012 ▶; Rakhshandeh et al., 2012 ▶). Briefly, aliquots of 10 µl of MTT solution (5 mg/ml) were added to culture medium and the reaction mixture incubated for 2 h. The mixture was removed and the resulting formazan was dissolved by adding 100 µl dimethyl sulfoxide to each well of the plate. The optical density of formazan dye was read at 570 and 620 nm (background) using a StatFAX303 plate reader. The percentage of viable cells was calculated as a ratio of optical density of treated to control (cultured in standard medium only) cells.

**Statistical analysis**

All data were expressed as mean±SEM. One way ANOVA followed by Tukey’s post hoc test for multiple comparisons were used for statistical evaluation. Statistical significance was accepted at p<0.05.

## Results

**Effect of individual agents on cell viability**

**Figure 1:**
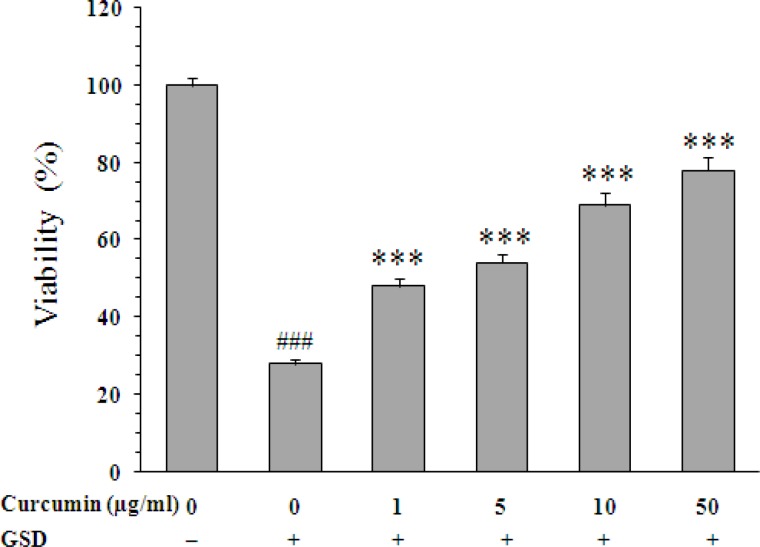
Effect of curcumin on viability of PC12 cells cultured for 8 h in the glucose/serum deprivation (GSD) condition. The bars show percentage of cell viability as compared with untreated cells cultured in the standard condition. Data are mean±SEM of n = 8 wells. *** p<0.001 *vs.* untreated cells cultured in GSD condition; ^###^ p<0.001 *vs.* cells cultured in the standard condition

**Figure 2:**
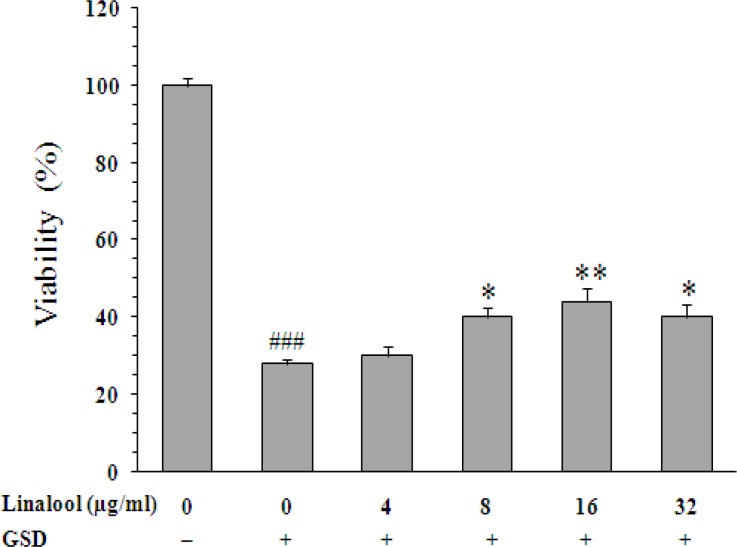
Effect of linalool on viability of PC12 cells cultured for 8 h in the glucose/serum deprivation (GSD) condition. The bars show percentage of cell viability as compared with untreated cells cultured in the standard condition. Data are mean±SEM of n = 8 wells. * p<0.05 and ** p<0.01 *vs.* untreated cells cultured in GSD condition; ^###^ p<0.001 vs. cells cultured in the standard condition

Exposure of PC12 cells to GSD condition led to 72% decrease on cell viability (28±1% *vs*. 100±2%, p<0.001). When the cells were exposed to 1, 5, 10, and 50 µg/ml of curcumin, percentage of cell viability increased from 28±1% (untreated cells) to 48±2.2% (p<0.001), 54±2.3% (p<0.001), 69±3.4% (p<0.001), and 78±3.4% (p<0.01), respectively (Figure 1). Moreover, linalool significantly increased the viability of cells at concentrations of 8 µg/ml (40±1.4%, p<0.05), 16 (44±1.4%, p<0.01), and 32 µg/ml (40±1%, p<0.05) as compared with untreated cells (Figure 2). 

**Figure 3:**
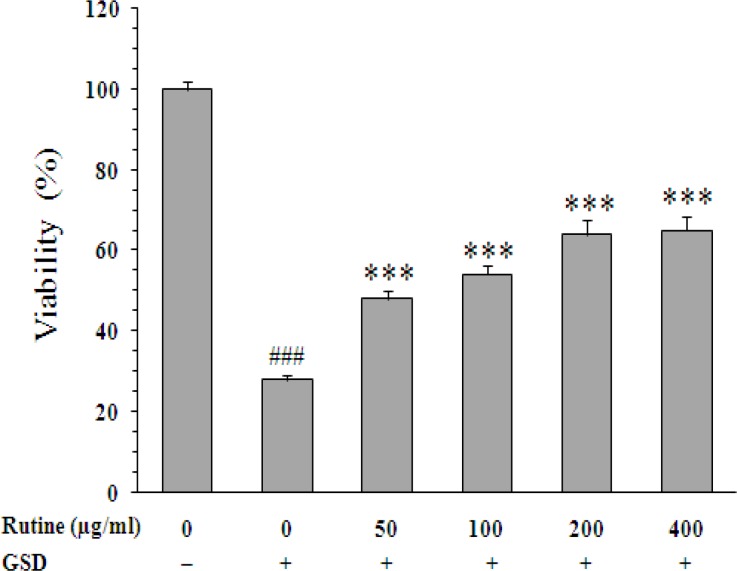
Effect of rutin on viability of PC12 cells cultured for 8 h in the glucose/serum deprivation (GSD) condition. The bars show percentage of cell viability as compared with untreated cells cultured in the standard condition. Data are mean±SEM of n = 8 wells. *** p<0.001 *vs.* untreated cells cultured in GSD condition; ^###^ p<0.001 *vs.* cells cultured in the standard condition

Effect of rutin on the cell viability is shown in Figure 3. When compared with untreated cells (28±1%), rutin at 50, 100, 200, and 400 µg/ml increased the surviving percentage to 48±1.5% (p<0.001), 54±1.7% (p<0.001), 64±0.5 (p<0.001), and 65±2.7% (p<0.001), respectively. 

**Figure 4:**
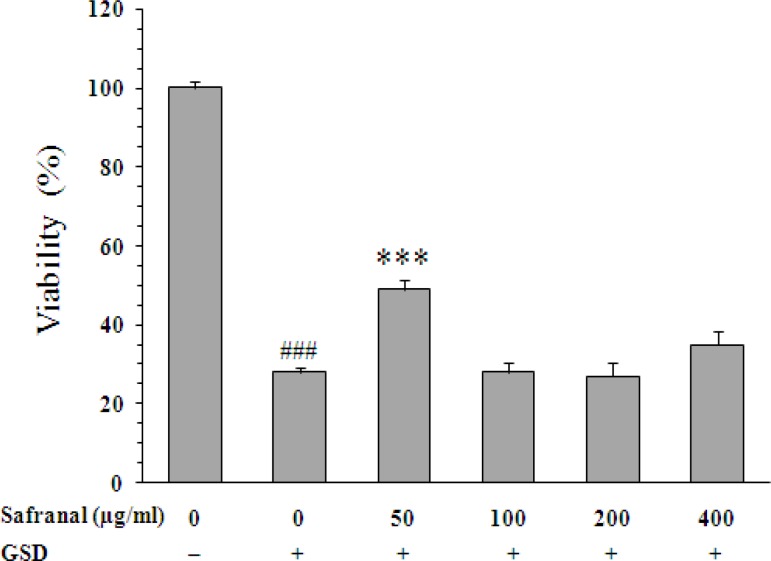
Effect of safranal on viability of PC12 cells cultured for 8 h in the glucose/serum deprivation (GSD) condition. The bars show percentage of cell viability as compared with untreated cells cultured in the standard condition. Data are mean±SEM of n = 8 wells. *** p<0.001 *vs.* untreated cells cultured in GSD condition; ^###^ p<0.001 *vs.* cells cultured in the standard condition

Similarly, the neurotoxic effect of GSD condition was inhibited by safranal (Figure 4). However, the effect of safranal was only observed at concentration of 50 µg/ml (49±2%, p<0.001).

**Figure 5:**
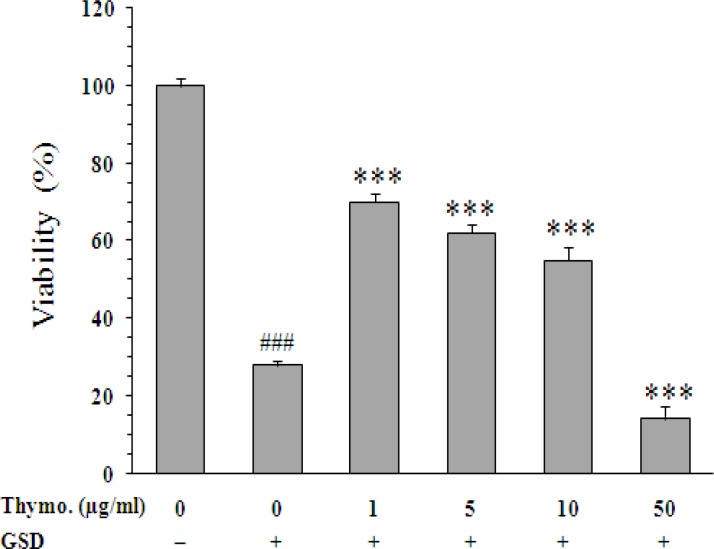
Effect of thymoquinone on viability of PC12 cells cultured for 8 h in the glucose/serum deprivation (GSD) condition. The bars show percentage of cell viability as compared with untreated cells cultured in the standard condition. Data are mean±SEM of n = 8 wells. *** p<0.001 *vs.* untreated cells cultured in GSD condition; ^###^ p<0.001 *vs.* cells cultured in the standard condition. Thymo: thymoquinone

As shown in Figure 5, exposure of the cells to low concentrations of thymoquinone leads to significant increase on cell viability. In the presence of 1, 5, and 10 µg/ml of thymoquinone, surviving percentage of the cells was 70±3.2%, 62±2.5%, and 55±10% (p<0.001 *vs.* untreated cells), respectively. Thymoquinone at concentration of 50 µg/ml not only failed to increase cell survival, but also further decreased the viability (14±0.5%, p<0.001 *vs.* untreated cells).

**Effect of combined agents on cell viability**

**Figure 6:**
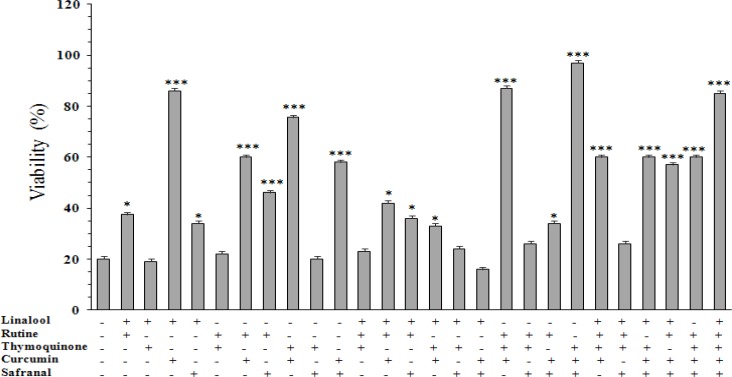
Effect of combinations of curcumin (10 µg/ml), linalool (16 µg/ml), rutin (200 µg/ml), safranal (50 µg/ml), and thymoquinone (1 µg/ml) on viability of PC12 cells cultured for 8 h in the glucose/serum deprivation (GSD) condition. The bars show percentage of cell viability as compared with untreated cells cultured in the standard condition (100±2%). Data are mean±SEM of n = 8 wells. * p<0.05 and *** p<0.001 *vs.* untreated cells cultured in GSD condition

Figure 6 shows the effect of combinations of curcumin (10 µg/ml), linalool (16 µg/ml), rutin (200 µg/ml), safranal (50 µg/ml), and thymoquinone (1 µg/ml) on GSD-induced cell death. When these agents were combined by pairs, the better cytoprotective activities were observed at these combinations linalool+curcumin (86±3.7%), thymoquinone+curcumin (76±5%), rutin+curcumin (60±2.8%), safranal+curcumin (58±2.3%), and rutin + safranal (46±6.2%).

The effect of all these combinations were statistically significant (p<0.001) compared with untreated cells (20±2%).When three agents were mixed, percentage of cell surviving increased from 20±2% (untreated cells) to 87±2% (p<0.001) and 97±5% (p<0.001) at these combinations rutin+curcumin+thymoquinone and safranal+curcumin+thymoquinone combinations, respectively. The combinations of four agents were also tested on the cell viability. Except for linalool+rutin+safranal+thymoquinone, all other combinations decreased (p<0.001) the GSD-induced cell death significantly.

The mixture of all agents, curcumin+linalool+rutin+safranal+thymoqui-none, was also able to increase cell viability under GSD condition (85±1.5%, p<0.001).

## Discussion

In this study, glucose and serum limitation-induced damage in PC12 cells were used to partially model the pathological process of cerebral ischemia in an attempt to search a phytochemical compound with potent neuroprotective effect. Our results showed that all the tested agents (curcumin, linalool, rutin, safranal, and thymoquinone) are able to inhibit neurotoxic effect of GSD condition. These results are in agreement with previous studies demonstrated neuroprotective effects of curcumin (Mendonca et al., 2012 ▶; Park et al., 2008 ▶; Rui et al., 2008 ▶), linalool (Batista et al., 2010 ▶; Devi et al., 2007 ▶; Elisabetsky et al., 1995 ▶), rutin (Khan et al., 2009 ▶; Pu et al., 2007 ▶), safranal (Fukui et al., 2011 ▶; Hosseinzadeh et al., 2005 ▶; Hosseinzadeh et al., 2007 ▶; Mousavi et al., 2010b ▶), and thymoquinone (Mousavi et al., 2010a ▶). Effects of curcumin and rutin were seen in a concentration-dependent manner. However, the best cytoprotective activities of safranal and thymoquinone were observed at low concentrations. It may be due to the cytotoxicity of high concentrations of safranal and thymoquinone (Abdullaev et al., 2003 ▶; Behravan et al., 2010 ▶; Khader et al., 2009 ▶).

We hypothesized that combinations of curcumin, linalool, rutin, safranal, and thymoquinone may have better neuroprotective effect. To test this thesis, all possible combinations of these agents, 26 mixtures, were exposed to the cells cultured in GSD condition. Most of the combinations could decrease the GSD-induced cell death. However, only linalool+curcumin, thymoquinone+curcumin, rutin+curcumin+thymoquinone, safranal+curcumin+thymoquinone, and curcumin+linalool+rutin+safranal+thymoquinone compounds were able to induce better neuroprotective effect than curcumin or thymoquinone which showed the best effect in monotherapy. Curcumin is a common constituent of these compounds and its combination with thymoquinone and safranal could restore the cell viability to the normal level. On the other hands, some mixtures (linalool+thymoquinone, rutin+thymoquinone, safranal+thymoquinone, linalool+rutin+thymoquinone, linalool+safranal+thymoquinone, curcumin+linalool+safranal, rutin+safranal+thymoquinone, and linalool+rutin+safranal+thymoquinone) not only failed to enhance cell viability, but also abolished the cytoprotective activities of each individual agent. Although it is difficult to draw a conclusion from such complex finds, this effect may be due to possible interaction between thymoquinone and other agents when curcumin is absent.

In conclusion, our study revealed that curcumin and thymoquinone have the best neuroprotective effects among the tested phytochemicals; a combination of safranal+curcumin+thymoquinone can block GSD-induced cell death. Therefore, confirming these results in *in vivo* studies, this combination has the potential to be used as a new therapeutics for cerebral ischemia and neurodegenerative diseases.
